# Mental health experiences and coping strategies of BAME care workers who worked in nursing and residential care homes during the COVID-19 pandemic in Luton, England

**DOI:** 10.1186/s12889-023-15423-2

**Published:** 2023-03-29

**Authors:** Isabella Kabasinguzi, Nasreen Ali, Peter Ochepo

**Affiliations:** Institute for Health Research, University of Bedfordshire, Putteridge Bury, Luton, Bedfordshire, LU2 8LE UK

**Keywords:** “Mental health”, “Care workers” Experiences, Coping, Strategies, COVID-19, Pandemic, Luton, Residential, Nursing, Home

## Abstract

**Background:**

The COVID-19 pandemic intensified the risk factors for poor mental health among care workers in the UK. However, there is inadequate evidence on the mental health impact of COVID-19 on Black, Asian, and minority ethnic (BAME) care workers in particular. This study seeks to explore mental health experiences and coping strategies of BAME care workers who worked in nursing and residential care homes during the COVID-19 pandemic.

**Method:**

This is a qualitative study conducted between February and May, 2021 in Luton, England. A sample of n = 15 care workers from BAME background working in nursing and residential care homes were recruited purposively using the snowball sampling technique. In-depth interviews were conducted around topics such as views on COVID-19, the impact of the COVID-19 pandemic on mental health and coping during the COVID-19 pandemic. Data from the interviews was analysed using the Framework Analysis Approach.

**Results:**

The COVID-19 pandemic had a negative impact on the participants’ mental health as they experienced stress, depression, anxiety, trauma and paranoia. The majority of the participants explained that they managed their mental health by belief in God and religious practices, by keeping themselves busy doing activities they were passionate about, following government guidelines on the prevention of COVID-19, seeing the service users happy and some participants managed through support that was offered by the government. However, some participants did not have any support for their mental health.

**Conclusion:**

Issues such as increased workload associated with COVID-19 restrictions engendered mental health problems among BAME care workers, however, the workload only further increased during the pandemic, but the health and social care sector was already affected by heavy workload due to staff shortages and this needs to be addressed through increasing their wages to encourage more people to work in the health and social care sector. In addition, some BAME care workers never received any support for their mental health during the pandemic. Hence, integrating mental health services such as counselling, supportive psychotherapy and recreational therapies in care homes could help to support the mental health of care workers in the COVID-19 era.

**Supplementary Information:**

The online version contains supplementary material available at 10.1186/s12889-023-15423-2.

## Background

The COVID-19 pandemic has had a detrimental impact on health and care workers globally [1]. According to the World Health Organisation (WHO), approximately 180,000 health and care workers died due to COVID-19 between January 2020 and May 2021 around the world [1]. England is one of the countries that reported the highest deaths among health and care workers worldwide [2]. By January 2021, over 850 health and care workers had died from COVID-19 in England and Wales [3]. Health and care workers have been at the frontline of caring for patients in England during the COVID-19 pandemic and associated lockdowns [4, 5]. They were therefore at higher risk of infection and transmission which has resulted to the loss of many health and care workers’ lives [6]. They have witnessed the loss of many service users’ lives [7–9] and consequently this may have had an impact on their mental health [10–12] During the COVID-19 Pandemic, health and care workers in the UK reported mental health issues such as acute stress, post-traumatic stress disorder, depression and anxiety [13].

The COVID-19 pandemic has had a significant impact on the mental health of care workers in particular [14]. It is worth noting that care work was already a stressful job due to work force shortages, unsecure contracts, unpaid travel time and work place exploitation [15]. It is argued that the COVID-19 pandemic exacerbated the situation [16] as it intensified the risk factors for poor mental health among care workers [17]. The pandemic presented a dramatic increase in workload due to having to isolate some service users, wearing PPE for very long hours and at times they were not able to provide the quality care they would normally provide [18]. Despite England having a population of approximately 840,000 care workers, around 27.8% of them leave their jobs each year because of the associated challenges [17, 19]. Although it was emphasised that fighting the COVID-19 pandemic was everyone’s business [20], the major burden of treating and caring for patients in the UK fell on the underfunded and understaffed health and social care sector [21]. In fact, the COVID-19 pandemic came when England was facing a health and care crisis [22–26]. It should be pointed out that 1 in 5 care workers in England are from BAME background [27]. There is increasing evidence that the COVID-19 pandemic has had a disproportionate effect on BAME groups in the UK [28–31]. An analysis of National Health Service (NHS) deaths in April 2020 revealed that 64% of COVID-19 related deaths among NHS staff were people from BAME background [32]. Approximately 50% of the NHS staff who died from COVID-19 were born outside the UK [33].

Furthermore, care workers from BAME groups were at higher risk of mental health issues due to the inequalities that existed among BAME groups before the pandemic [34]. A survey conducted by MIND in 2020, affirms that the inequalities that existed among BAME groups worsened their mental health during the pandemic, for instance, concerns about finances made the mental health of 50% of people from BAME groups worse compared to 45% of those from white groups [35]. Additionally there were notable inequalities in access to mental health services among people from BAME groups in the UK even before the current pandemic [34]. Evidence shows that people from BAME groups receive worse mental health care than people from the majority group [36]. Findings of a review by BMJ show that people from BAME groups were less likely to access support for their mental health in primary care through their General Practitioner (GP) and were more likely to find themselves in crisis care [35]. More studies show that there have been very low rates of access to mental health services such as psychological therapies among people from BAME background [37–39]. Literature suggests that workers in the health and care sector coped with their mental health through psychological support, belief in God, relaxation, family segmentation, work and accessing Personal Protective Equipment (PPE) during the COVID-19 pandemic [40–42]. However, little is known about the coping strategies of BAME care workers in particular. In addition, the, UK government responded to the COVID-19 mental health impact through implementing The COVID-19 Mental Health and Wellbeing Recovery Action plan [43]. Providentially, the NHS Mental Health Implementation Plan 2019/20–2023/24 was already being implemented [44]. Though England has achieved considerable success in the provision of Perinatal Mental Health, Early Intervention in Psychosis and improved mental health data tracking [45], literature reveals that mental health services are still a long way behind most physical health services in terms of their resourcing, the ability of patients to access them and patient outcomes [45, 46]. For instance in 2021, NHS England invested £15 million in to supporting the mental health of their staff, however, care workers who did not work directly with the NHS were not part of this support yet they are essential workers and they provided care to COVID-19 patients in nursing and residential care homes during the pandemic [47]. Moreover, numerous studies have been conducted on the impact of the COVID-19 pandemic on the mental health of frontline workers but there is inadequate evidence on the impact of COVID-19 on the mental health of BAME care workers who work in nursing and residential care homes [48–50].

The aim of this qualitative study is to explore BAME care workers’ mental health experiences and coping strategies during the COVID-19 pandemic in Luton, England. This study contributes to the existing evidence base on the mental health impact of COVID-19 on care workers. An in-depth understanding of their experiences is essential for designing interventions that can help to address the mental health issues in the COVID-19 era.

.

## Method

This study was carried out between February and May 2021, in Luton, a town located in the south east of England [51]. Luton is a diverse town consisting of a high number of the BAME groups [52] who are most likely to work in the care sector as they make up 1 in every 5 people who work in adult social care in England [12]. In 2011, Luton was made up of over 50,200 Asians and the black population was up to 19,800 [52]. In addition, there are nursing and residential care homes in and around Luton [53]. Care workers from nursing and residential care homes were recruited purposively using the snowball sampling technique [54]. The first seven care workers were approached and requested to recommend other care workers who lived in Luton. The inclusion and exclusion criterion was used to include only care workers who lived in Luton, working in nursing and residential care homes during the COVID-19 pandemic. All the eligible care workers who were included in the study were categorised based on age, ethnicity, gender, marital status, education level and religion making sure that all the categories were represented. The care workers were sent participant information forms, consent forms and a zoom link invitation to attend one-to-one in-depth interviews that lasted around one hour. The interviews were carried out using a semi-structured discussion guide which was developed basing on current literature related to the impact of COVID-19 on the mental health of BAME care workers [55–61]. It focused on the following main themes: views on COVID-19, the impact of the COVID-19 pandemic on mental health, and managing during the COVID-19 pandemic. The interview discussion guide was made up of questions such as (a) what do you know about COVID-19?, (b) How has COVID-19 impacted on your mental health? (c) Give me examples of situations that have distressed you during the COVID-19 pandemic? (d) What was your greatest worry during the COVID-19 pandemic? (e) How did you manage during the COVID-19 pandemic? (f) What support did you have to cope during the COVID-19 pandemic? The interviews were conducted by the authors in person. They were conducted in English and then audio recorded.

### Data analysis

The Framework Analysis Approach was used to analyse the data [62]. Framework analysis is a comparative type of thematic analysis which uses an organised structure of inductively- and deductively-derived themes to carry out cross-sectional analysis using a combination of data abstraction and description [63] Framework analysis involves data farmiliarisation, developing a framework, coding, data extraction and interpretation [62]. Frame work analysis is ideal for analysing data in which participants tell their story rather than just answering questions.This study employed a semistructured discussion guide which enabled the participants to tell their experiences, making it the most suitable method of analysis for it [64]. Firstly, all interview audios were subsequently transcribed and thoroughly read to identify a list of recurrent themes, subthemes and key ideas from the transcripts. Secondly, the recurrent themes and subthemes that were identified in the data were used as codes to formulate a frame work. The coding was done by one corder. And the frame work contained three columns; the first one representing themes, the second representing sub-themes referred to as codes and the last representing verbatim from transcripts. Thirdly, all the transcripts were annotated with codes from the framework. These were supported by text descriptors such as stress, depression, anxiety, trauma, paranoia, God, busy, Personal Protective Equipments (PPE), resident’s welfare as elaborating the codes according to the framework and this was applied to every interview transcript. Fourthly, after annotating the transcripts with codes, the data that was coded was then extracted from the transcripts to the framework. And finally concepts, relationships and explanations from the data were established and presented.

## Results

The characteristics of the participants are presented in Table 1. These include age, sex, marital status, ethnicity, religion and level of education. The main themes that emerged from the analysis were: the impact of COVID-19 on BAME care workers’ mental health, and coping during the COVID-19 pandemic.

**Table 1:** self-reported participant bio- characteristics

Age	sex	Marital status	Ethnicity	Religion	Level of education
60% 20-30years	53% Female	60% Single	86% Black African	87% Christians	67% Master’s degree
26% 30-40years	47% Male	40% Married	13% Asian	13% Moslems	20% Undergraduate
7% 40–50 years					13% Secondary education
7% 50–60 years					

### The impact of COVID-19 on BAME care workers’ mental health

Nearly all the participants reported that the COVID-19 pandemic had a negative impact on their mental health. Most of the participants mentioned that stress was a key mental health issue they experienced during the pandemic. They stated that following government guidelines led to an increase in their workload. They were required to wear and change PPE from time to time, this meant additional work for them and consequently engendered stress. : “*Where on a normal day, it would have been a group thing which would have been done like on a table, it now required you to go from room to room, wearing of PPE, disposing it and going to another room wear PPE again. So it was a lot of stress.” (Male care worker aged 20–30)*.

Most of the participants considered wearing masks for very long hours to have brought about stress. They expressed that even on night shifts that lasted around 12 hours they were expected to keep their masks on as long as they were at work which was very stressful as it is not something they were used to. *“I have got a lot of stress especially due to the PPEs; especially the mask. You know you being in a mask twenty four seven you know like it causes shortness of breath for me I really can’t breathe through the nose mask all night. And the people that you are working with want you to put it on like twenty four hours.”(Male care worker aged 20–30)*.

A few participants considered helping the service users to adhere to the government guidelines to be very stressful. They stated that they spent a lot of time helping the service users to follow the guidelines which most of the service users failed to do and this stressed the participants as it further increased their workload, having to wipe all the surfaces numerous times. “*As simple as we were trying to have them sanitize their hands, it was difficult for us. so, it used to stress me out a lot because every time they went out, especially to wash their hands, use sanitizer, they wouldn’t, so, I would try to get a wipe and something and start wiping, so, I was constantly annoyed and trying to stay on top of things so I was trying to wait and keep all sorts of things clean. I just felt so stressed although that is part of my work.”(Female care worker aged 30–40)*.

Some participants reported that they went through depression during the COVID-19 pandemic. They substantiated that they were lonely during the pandemic.They had no people to physically interact with after work and even their interaction with others at work was limited due to the COVID-19 restrictions .To some of them, the loneliness progressed to depression.:*“I felt depressed because at times I would want someone to talk to, see a friend to discuss one or two things but no one to discuss with”(Male care worker aged 30–40)*.

Some of the participants reported that the fear of getting infected with the corona virus brought about anxiety. They stated that at some point almost all their work colleagues had been infected with the virus and this made them think that they were going to catch the virus anytime even though they were trying their best to protect themselves. This feeling was triggered by the fact that almost all their colleagues had tested positive despite protecting themselves from the virus. This led to an increase in their anxiety. :*“you don’t know whether as much as you are trying your best to protect yourself, there is still panic that somehow you are still going to contract the virus in spite of doing your own best to protect yourself. So, there has been anxiety in that aspect. In a case you commute in public transport and every day you commute, you mix with different people and also at work so you just really, so people say that they did their best but still they contracted the virus all the same so you get anxious in spite of doing everything you still might not escape the virus”.(Female care worker aged 30–40)*.

Few participants reported that they were traumatised by seeing very many people die every day due to COVID-19. They reported that they would go to work expecting more than five people to die, which was very traumatising. They enunciated that even those people who seemed to be fine would die in a short time. “*When you go to work, you would go expecting to lose five people on the same day; that was horrible to see. When you see someone fine and the next minute when you go to their room you find the person is already gone”.(Female care worker aged 30–40)*.

Some participants also reported that they experienced paranoia during the COVID-19 Pandemic. While some participants expressed that working with a COVID-19 patient for the first time left them paranoid, others reported that losing a service user they had supported very many times made them become paranoid. “*So when he suddenly passed away, it kind of made me feel very sad and it kind of made me ten times more conscious and paranoid in terms of like I am even more aware now. I was doing night shift by myself like I was the only one in the entire building at that time so it was a bit scary. So my greatest fear then at that time my greatest fear was literally having COVID-19.”(Female care worker aged 20–30)*.

### Coping during the COVID-19 pandemic

The majority of the participants reported ways through which they managed the mental health challenges that the COVID-19 pandemic presented. Most of the participants considered belief in God and associated religious practices as an effective way through which they managed during the pandemic. For instance, some participants stated that their belief in God helped them to manage during the pandemic as they were strengthened through reading the bible. They considered relying on God to be very important and a strong pillar in their ability to cope during the pandemic. “*You know I believe in God, so I was being strengthened by that. I was reading the bible and the word of the bible was very encouraging and reminding me that God is with me.”(Female care worker aged 20–30)*.

Some participants narrated that keeping themselves busy doing the things they were passionate about during the pandemic helped them to cope. They reported that keeping busy kept their minds focused on other things instead of focusing on what was happening in relation to COVID-19 especially the things that engendered anxiety. They discussed that spending time doing the things they loved helped them not to focus on the pandemic but on the things they loved and this kept them moving despite everything they were going through. While some participants focused on writing, others focused on developing their businesses. “*I got myself very busy with research and I decided to start my research about my business. It didn’t even let me think of what is going on in the world because when I am doing hair or make up I am in the moment and I love it.”(Female care worker aged 20–30)*.

Some of the participants averred that following government guidelines on prevention of COVID-19 helped them to manage during the pandemic. They mentioned that following government guidelines enabled them to continue working as they knew that they were protected. The guidelines gave them an assurance to continue caring for vulnerable people safely without fear of spreading the virus to them or contracting the virus. “*What I did was following the guidelines of NHS. The fact that I take care for people, I still wanted to care for them. So, I was just washing my hands, wearing my PPE, using my sanitizer, and all that” (Female care worker aged 20–30)*.

Some participants mentioned that seeing service users smiling and happy enabled them to cope during the COVID-19 pandemic. They stated that the happiness of their service users was very important to them as it motivated them to continue working regardless of all the challenges because of they cared about the happiness of service users. Some participants explained that the service users relied on them and if they gave up, the service users would have no one to rely on for support. They reported that the importance of their service users’ welfare helped them to cope. “*Seeing the service user’s smile was a major thing that happened and made me happy. A resident that had dementia, even when I took some weeks without working there she could remember my name and that meant a lot. Sometimes I would go to their rooms and play chase with someone who had COVID-19, just to be with the person. Sometimes I could go in to their rooms to see them smiling, see them laughing through it all, it was a very good coping strategy; it takes stress off.”(Male care worker aged 20–30)*.

Some participants reported that they had a form of support that helped them to cope during the COVID-19 pandemic. Some participants stated that they received support from the government which helped them to cope. They mentioned that government schemes such as bonuses kept them happy and lively and prevented them from crumbling in the face of COVID-19. “*There were also a lot of bonuses which of course trust me when you get bonuses, you feel very excited, Just being lively really because the more you are sad and down and worried about everything that is happening; The likelihood of you crumbling in the face of adversity.”(Female care worker aged 20–30)*.

However a few participants stated that they never received any support from anyone to help them cope during the COVID-19 pandemic. *“I didn’t get any support really to be quite honest, financially or emotionally that this support is dedicated to you because you went through trauma from COVID-19.”(Male care worker aged 50–60)*.

## Discussion

This is the first study to explore BAME care workers’ mental health experiences and coping strategies during the COVID-19 pandemic in Luton. The findings of this study contribute to the existing literature on the impact of the COVID-19 pandemic on the mental health of care workers and their coping strategies. The main themes that emerged from the analysis include; the impact of COVID-19 on care workers mental health, and coping during the COVID-19 pandemic. Stress was a key mental health issue that the majority of the participants experienced during the COVID-19 pandemic. This was due to an increase in workload;wearing PPE regularly and helping the service users to adhere to COVID-19 prevention measures meant extra work for the participants and this brought about stress. This correlates with a study which found that an increase in workload related to COVID-19 impacted on the mental health of healthcare workers [18]. It is worth noting that though wearing the PPE was considered stressful; it was one of the most effective ways through which the participants could protect themselves and the people they were supporting from contracting COVID-19. As much as it was stressful for the majority of the participants to wear PPE, it enabled some participants to manage as they explained that it gave them assurance that they were protected from COVID-19. This resonates with some existing studies on the mental health of healthcare workers during the COVID-19 pandemic [65, 66].

Depression was also experienced by some participants, it aggravated from loneliness due to the COVID-19 lockdown and less interaction at work. Mention should be made that 80% of the participants were Non-British nationals and therefore never had family to go back home to or even physically talk to during the COVID-19 pandemic. They mainly talked to family and friends through the phone and some participants stated that they needed someone to physically interact with. This explains why their loneliness aggravated to depression. These findings are in line with other studies which found that lack of physical interaction has a strong association with depression [67, 68].

Furthermore, the fear of getting infected with corona virus due to working in areas of high infectivity rates brought about anxiety among the majority of the participants. It should be pointed out that BAME care workers worked in areas of high infectivity [32]. Many people in care homes were being infected with COVID-19 and dying, these included their work colleagues and the service users. In addition, some studies have found that there was discriminatory deployment of BAME staff in potentially high virus exposure [32, 69] which accounts for their anxiety.

Religion and belief in God was a significant coping strategy to most of the participants. They stated that their belief in God strengthened them regardless of the challenges presented by the COVID-19 pandemic. It should be born in mind that all the participants belonged to a religion. This correlates with a study which found that religion and belief in God were central in coping among BAME communities [70]. A study on faith in God and response to mental health treatment revealed that belief in God was higher among the participants who responded to treatment compared to those who did not respond to treatment. This study concluded that belief in God was associated with better treatment outcomes [71].

Support from the government was essential in helping some participants to cope during the COVID-19 pandemic. Some participants mentioned that they received bonuses which motivated them and kept them going. These findings coincide with existing evidence on government support helping people to cope during the COVID-19 pandemic [72, 73]. However, some participants never received any support yet they needed it to help them cope during the pandemic. Interestingly though support from the government was provided, it was majorly financial support that was provided to them. Hence there is need for more efforts to be directed towards supporting the mental health of care workers as they also worked on the frontline during the pandemic and were considered essential workers.

### Limitations

This study had some limitations. Considering the fact that a non-random method was used to select the study participants, not every care worker in Luton had an equal chance of taking part in the study or being part of the study sample. This indicates that the study was prone to selection bias. In addition, the findings of this study cannot be generalized to care workers in other parts of England as it only used a sample of care workers from Luton. The circumstances surrounding care workers might be different from those in other parts of England hence influencing the way they experience the impact of COVID-19 and their coping strategies.

This study took place at a specific point in time during the pandemic, and so illustrates experiences during a particular point of the pandemic, rather than the pandemic as a whole, which is still on-going. The fact that there was one coder, they might have been limited by their subjectivity.

## Conclusion

The COVID-19 pandemic had a negative impact on the mental health of BAME care workers who worked in nursing and residential care homes in Luton. An increase in workload associated with government guidelines on the prevention of COVID-19, having no one to physically interact with, and working in areas of high infectivity rates engendered mental health issues such as stress, depression and anxiety among BAME care workers during the COVID-19 pandemic. The majority of the care workers managed their mental health by belief in God and associated religious practices, some care workers managed through wearing PPE, seeing the service users happy, keeping busy doing the things they were passionate about and receiving financial support from the government in form of bonuses. It is important to note that workload only further increased during the pandemic, but the health and social care sector was already affected by heavy workload due to staff shortages and this needs to be addressed by increasing their wages to encourage more people to work in the health and social care sector. In addition, some BAME care workers never received any support for their mental health during the pandemic and some of them never had family to physically talk to. Interestingly even those who received support from the government, it was mainly financial support that was provided to them. Hence, integrating mental health services such as counselling, supportive psychotherapy and recreational therapies, in care homes could help to support the mental health of care workers and build their resilience in the COVID-19 era.

## Electronic supplementary material

Below is the link to the electronic supplementary material.

Supplementary Material 1

## Data Availability

All the data generated or analysed during the study are included in this published article and its supplementary files.
