# Supplementary material for: Mental health experiences and coping strategies of BAME care workers who worked in nursing and residential care homes during the COVID-19 pandemic in Luton, England
Source: BMC Public Health. 2023 Mar 29;23:592. doi: 10.1186/s12889-023-15423-2 (PMC10054189; doi:10.1186/s12889-023-15423-2)
Supplement: Supplementary file 1 — Supplementary Material 1 [file 12889_2023_15423_MOESM1_ESM.docx]

**Coding framework for mental health experiences and coping strategies of BAME care workers who worked in nursing and residential care homes during the COVID-19 pandemic in Luton, England.**

| **Themes** | **Subthemes** | **Verbatim** |
| --- | --- | --- |
| The impact of COVID-19 on BAME care workers’ mental health | Stress | “Where on a normal day, it would have been a group thing which would have been done like on a table, it now required you to go from room to room, wearing of PPE, disposing it and going to another room wear PPE again. So it was a lot of stress.”  “I have got a lot of stress especially due to the PPEs; especially the mask. You know you being in a mask twenty four seven you know like it causes shortness of breath for me I really can’t breathe through the nose mask all night. And the people that you are working with want you to put it on like twenty four hours.”  “In most cases you have to put on the essential PPE and all of that, it is as well stress; you have to wear it and change which is stress. it is something you are not used to, you have to wear PPE and you must do it because if you do not do it that means you do not have a job to do so you can’t eat.”  “Having to get your service user to adhere to these particular guidelines… it was a stress of its own.”  “The major situation that kind of stressed me was when there was an outbreak in a care home, I was working in and the residents had to stay in their rooms and we had to go to their rooms to give them personal care and individual needs. Where on a normal day, it would have been a group thing which would have been done like on a table but now you had to go from room to room, wearing of PPE, disposing it and going to another room wear PPE again. So it was a lot of stress, sweating under the PPE but you cannot take it off.”  “Stress in a sense that we all work, work, work whereby there is no time for relaxation**.”**  “you know another stress is in conducting the tests, imagine dipping the thing into your nose, I won’t say the mouth, into the nostril it becomes tiring but as a way of preventing it and checking it is also a cost.”  “I mean it got a bit boring to go from your home to work place and from your work place to your home straight without being able to visit anywhere even in your holidays so it was a bit stressful”  “It was very stressful because there was no one else to do it.”  “So we had to stay for like fourteen hours every day. So it was quite draining.”  “Actually it made me feel like I was a washing machine, like you are literally just using me until I become positive. And because I wasn’t positive, I was being used.”  **“**Staff declined shifts, so the few staff that came to work would always have more workload to do.”  “There was increase in workload because of course there were new rules, you have to follow each and every rule like when you are going to the residents you have to put on full PPE something that you were not doing before but now this is a new rule that has come in.”  “When any staff was positive all the other staff they have been in contact with would not be able to work for the next 1o days so due to that you who are available because I am an agency worker, the people who are available would have to work more than before.” |
|  | Depression | “I felt depressed because at times I would want someone to talk to, see a friend to discuss one or two things but no one to discuss with”  “…depression any time any day, you know you could be at work, and it sets in and definitely it has to affect your work, your output, your capacity it has to affect it, you know sometimes you feel like you are tired of everything and that suddenly causes destruction but we still try to carry on”  “Depression here and there comes and goes, though not clinical because sometimes you get down, you know, social interaction is very healthy for mental health. So, when they take that health from you, they take something away from you.”  “I had a few people that went through depression and anxiety because firstly they are at high risk, they have breathing difficulty and underlying illness, so because they had to isolate by themselves and also having no means of either accurate firm being or firm interactions, they become very depressed.”  “Like I was mentally in a prison and although it was a nice relaxing time, but just because someone said “you must stay home”, it is a law, you must stay home, it kind of brought mental like bondage in your head.”  “All of a sudden I stopped seeing people even when you go to the shop and sneeze everybody runs away from you. Whatever you do, people run away from you. Your life is kind of regimented.”  “If you are a single person like us you could not even go out to meet your date. You cannot compare having a phone date to having a physical date.” |
|  | Anxiety | “you don’t know whether as much as you are trying your best to protect yourself, there is still panic that somehow you are still going to contract the virus in spite of doing your own best to protect yourself. So, there has been anxiety in that aspect. In a case you commute in public transport and every day you commute, you mix with different people and also at work so you just really, so people say that they did their best but still they contracted the virus all the same so you get anxious in spite of doing everything you still might not escape the virus”  “I think for me it was anxiety because I was very anxious. Every morning when I woke up and wanted to go to work I was very anxious”  “When COVID-19 started and where we were working there was no PPE, the only thing we were using that time was just hand sanitizer, people were scared and in the same area I was very anxious to take care of patients”  “So one minute I felt like ; yeah I definitely don’t have it , you know and the next minute I am thinking that “I might have it, I am sneezing now.” and also I was also contact with a few of my friends that were nurses. So it was like everywhere I go, it became very scary I am not going lie.”  **“…** I did a night shift when he died. So I think that kind of put like a bit anxiety perhaps!”  **“….** me I was much tensed, it was very tough for me especially.”  “The fact that you get called and literally I felt like it was a threatening call from the government. Like they would call you and say “have you been outside” I felt so dehumanized.”  “If I am to have it, it’s going be worse.” Like I am going to have breathing difficulty you know that it will be very intense so that was my biggest worry.”  “Yeah, it gave me a lot of thoughts. Like when I wake up or before I leave my bed in the morning to go to work, I am scared but I just can’t help it, yeah, if we all abandoned these guys no one would take care of them so we just had to do our best and find ways of preventing us from catching it or from contracting it.”  “At the early stage, during the early stage it was just like a movie. You know lockdown everywhere bla, bla. So I was beginning to say ohh my God!!! What is this? Is this going to end? I began also ask myself “I’m I going to cope with this whole thing especially with the lockdown.”  **“** I have been affected to find out that people around me, the people I had known passed away. I knew quite a few people that I knew directly and passed away because of the corona virus.” |
|  | Trauma | “When you go to work, you would go expecting to lose five people on the same day; that was horrible to see. When you see someone fine and the next minute when you go to their room you find the person is already gone.”  “It became so, so traumatizing”  “It became so, so traumatizing even affected the studies a lot and also recreational activities like the gym and the other things people normally go for social life and development so it was just all about home and work**.** Life last year was just stagnant to be honest. So it affected deeply.”  It felt like I was in some kind of bondage. |
|  | Paranoia | “So when he suddenly passed away, it kind of made me feel very sad and it kind of made me ten times more conscious and paranoid in terms of like I am even more aware now. I was doing night shift by myself like I was the only one in the entire building at that time so it was a bit scary. So my greatest fear then at that time my greatest fear was literally having COVID-19.”  “It made me paranoid, when it started earlier; I remember my first shift to be working with a corona virus patient at that time it was quite scary.”  “I was paranoid due to the corona virus that was going on or that is still going on but that did not deter me from doing what I am there for. I am there to care for the residents.”  “We were being conscious of everything we do, and suspecting ourselves. Everyone was a suspect which never used to be.”  “I was doing night shift by myself like I was the only one in the entire building at that time so it was a bit scary. So my greatest fear then at that time my greatest fear was literally having COVID-19.”  Many people were dying, personally for me I was very scared because where I normally work, my field. I was very scared as I did not know what would happen tomorrow. You do not know whether you will have it or you will not have it, whether the person you are working with has it, you know. You have got family back home and you are coming back home.” |
| Coping during the COVID-19 pandemic | Belief in God and religious practices | “You know I believe in God, so I was being strengthened by that. I was reading the bible and the word of the bible was very encouraging and reminding me that God is with me.”  “It is hard but for people who know God, by the grace of God some of us were able to cope during that period.”  “Actually, you can never do anything without God. So, relying on Him is very-very important for one no matter what time. So, I know that if God is by your side, nobody will be against you. So, because of that I was able to cope.”  “I am still today one of the luckiest people that are still negative which is unexplainable but I know that it’s God.”  “Also something that kept me going was the fact that I was attending fellowship, as well, of course not interacting, and social distancing but of course I was being strengthened by my belief. You know I believe in God, so I was being strengthened by that. I was reading the bible and the word of the bible was very encouraging and reminding me that God is with me.”  “I held on to the word of God and there sometimes when you hold on to what you believe in it has to manifest within yourself so because of that I could not be depressed, because I had something to rely on.”  “I haven’t understood how I did not get COVID-19 at some point because I don’t know but of course it was God because I had not had COVID-19 at a certain point when everyone else had it.”  **“**And trusting God that I would be okay and which of course I am.” |
|  | Keeping busy doing the activities they were passionate about | “I got myself very busy with research and I decided to start my research about my business. It didn’t even let me think of what is going on in the world because when I am doing hair or make up I am in the moment and I love it.”  “I was engaged in all the extra co- curricular activities that hindered me from even having to think of getting depressed.”  “Lately we are working too much! Too much! and that also keeps my mind apart from anything COVID like I really don’t pay attention to it anymore, you know because am busy, my mind is busy and am very occupied now. So being busy has also helped me a lot.”  “I kept myself busy producing and writing articles to be published. I was actually busy then; I was busy and busy for myself.” |
|  | Following government guidelines | “What I did was following the guidelines of NHS. The fact that I take care for people, I still wanted to care for them. So, I was just washing my hands, wearing my PPE, using my sanitizer, and all that”  “I even bought a pack of masks; everywhere I go I’m equipping myself with a mask, a hand sanitizer, gloves, and the apron things like that everywhere I go.”  “I think the best thing was just to follow the government guidelines.”  “…using PPE’s, I even bought a pack of mask, everywhere I go am equipping myself with a mask, a hand sanitizer, gloves, the apron things like that everywhere I go.”  “I made sure like I said before if you adhere to the strict guidelines it is very difficult for you to contact it.”  “I just placed my mind on this COVID thing has come to stay and all I have to do is be extremely careful so that I don’t get it.” |
|  | Seeing service users happy | “Seeing the service users’ smile was a major thing that happened and made me happy. A resident that had dementia, even when I took some weeks without working there she could remember my name and that meant a lot. Sometimes I would go to their rooms and play chase with someone who had COVID-19, just to be with the person. Sometimes I could go in to their rooms to see them smiling, see them laughing through it all, it was a very good coping strategy; it takes stress off.”  “The more they are happy, the more we feel better that we have provided good services.”  “I wanted to stop working but now with that these people are human beings and there is no body to take care of them-they need to be assisted they need to be supported. So, there is no other thing than to go ahead and support them.. |
|  | Support from family and friends | You have friends that would keep calling to make sure you are good and to make sure your mental health is intact but imagine those that do not even have friends they were very lonely and you had to like lock your self-indoor, just you, no contact with family and friends.  “The contact with friends and family, I do not think they helped much.” |
|  | Support from the government | “There were also a lot of bonuses which of course trust me when you get bonuses, you feel very excited, Just being lively really because the more you are sad and down and worried about everything that is happening; The likelihood of you crumbling in the face of adversity.”  “It was a government furlough; so that is the only support I know I got then.”  “I have remembered! The council actually gave me 500 pounds for isolating (laughs) that was quite encouraging (laughs).”  “I didn’t get any support really to be quite honest, financially or emotionally that this support is dedicated to you because you went through trauma from COVID-19.” |
